# Improved Multi-Stream Convolutional Block Attention Module for sEMG-Based Gesture Recognition

**DOI:** 10.3389/fbioe.2022.909023

**Published:** 2022-06-07

**Authors:** Shudi Wang, Li Huang, Du Jiang, Ying Sun, Guozhang Jiang, Jun Li, Cejing Zou, Hanwen Fan, Yuanmin Xie, Hegen Xiong, Baojia Chen

**Affiliations:** ^1^ Key Laboratory of Metallurgical Equipment and Control Technology of Ministry of Education, Wuhan University of Science and Technology, Wuhan, China; ^2^ Research Center for Biomimetic Robot and Intelligent Measurement and Control, Wuhan University of Science and Technology, Wuhan, China; ^3^ College of Computer Science and Technology, Wuhan University of Science and Technology, Wuhan, China; ^4^ Hubei Province Key Laboratory of Intelligent Information Processing and Real-time Industrial System, Wuhan University of Science and Technology, Wuhan, China; ^5^ Hubei Key Laboratory of Mechanical Transmission and Manufacturing Engineering, Wuhan University of Science and Technology, Wuhan, China; ^6^ Hubei Key Laboratory of Hydroelectric Machinery Design and Maintenance, China Three Gorges University, Yichang, China

**Keywords:** sEMG signals, gesture recognition, attention mechanisms, neural networks, multi-stream

## Abstract

As a key technology for the non-invasive human-machine interface that has received much attention in the industry and academia, surface EMG (sEMG) signals display great potential and advantages in the field of human-machine collaboration. Currently, gesture recognition based on sEMG signals suffers from inadequate feature extraction, difficulty in distinguishing similar gestures, and low accuracy of multi-gesture recognition. To solve these problems a new sEMG gesture recognition network called Multi-stream Convolutional Block Attention Module-Gate Recurrent Unit (MCBAM-GRU) is proposed, which is based on sEMG signals. The network is a multi-stream attention network formed by embedding a GRU module based on CBAM. Fusing sEMG and ACC signals further improves the accuracy of gesture action recognition. The experimental results show that the proposed method obtains excellent performance on dataset collected in this paper with the recognition accuracies of 94.1%, achieving advanced performance with accuracy of 89.7% on the Ninapro DB1 dataset. The system has high accuracy in classifying 52 kinds of different gestures, and the delay is less than 300 ms, showing excellent performance in terms of real-time human-computer interaction and flexibility of manipulator control.

## Introduction

When the number of patients with physical disabilities is on the rise (Chen et al., 2021a; Chen et al., 2022a), dexterous hand devices are not fully satisfying the needs of patients. So it is extremely important to design a dexterous hand control system to help patients with forearm disabilities restore some of their limb functions and improve their quality of life (Ma et al., 2020; Xie et al., 2020). As a physiological signal closely related to human movement, the EMG signal can intuitively reflect the user’s intention; and the EMG signal-based dexterous hand control system (Li et al., 2020; Yun et al., 2022; Xu et al., 2022) has received widespread attention. Because it is simple, safe to operate, and not susceptible to environmental influences such as light or environmental sound changes (Li et al., 2019a). There are two main approaches to obtain EMG signals: one uses needle electrodes to invade the body and obtain physiological signals directly. The other is to analyze the user’s movement status by placing electrodes to detect changes on the skin surface currently. Compared with the use of needle electrodes, sEMG has the advantages of non-invasive, painless measurement, easy acceptance by the subject, and simple operation. So it has been widely used in practice. For patients with hand disabilities, some forearm muscles remain at the residual limb, and their central nervous system is not damaged and can function normally.

However, due to the characteristics of very weak and noisy EMG signals, the effective recognition of EMG signals still needs further improvement (Jiang et al., 2019a; He et al., 2019; Tan et al., 2020; Yu et al., 2020). At present, the gesture recognition methods of EMG signals are mainly divided into traditional machine learning based and deep learning based (Lenz et al., 2015; He et al., 2016; Cheng et al., 2021; Duan et al., 2021); the traditional method consists of three parts, pre-processing (such as denoising and filtering), feature extraction, and classifier model classification. However, manual extraction of features and then classification is tedious, and the accuracy is not very satisfactory.

Deep learning is a method that requires massive data for experimentation. By pre-processing the initial signal and expanding the experimental data, researchers continuously optimize and improve various parameters in the deep learning model, repeatedly train the model using Convolutional Neural Network (CNN) (Weng et al., 2021; Tao et al., 2022), and continuously test to get the optimal experimental results, thus improving the recognition accuracy. At present, the deep learning model has made some progress in sEMG gesture recognition, but the accuracy is not high while ensuring real-time. the model’s ability to fit multi-gesture sparse EMG signal data and extract features need to be further improved. The existing CNN-based EMG gesture recognition research does not make full use of the timing information of sEMG signal data, and difficult to apply in bionic hand control systems.

To solve the above problems, a multi-stream fusion network (Zhang et al., 2022) of one-dimensional convolutional neural network (CNN) + GRU is proposed, which embeds the attention mechanism (CBAM) in the CNN . CNN)+ GRU is used to for processing to extract the hidden correlation characteristics between the sEMG sequence signals, and embeds an attention module to learn synergy of different sEMG feature channels, and the spatiotemporal features. At the same time, ACC signals are added for recognition to further improve the accuracy. Based on this network, a dexterous hand control system is established to classify the collected sEMG signals and control the bionic hand to do matching movements according to the user’s intention, which can assist people with hand disabilities to live normally (Li et al., 2022; Tao et al., 2022). The contributions of this paper are as follows.

1) Acceleration signals and sEMG signals were collected to construct a dataset containing 52 different hand gestures.
2) Embedding CBAM units in a 1D convolutional network selectively emphasize informative features and suppress useless features on channels and spaces, enhancing the effective extraction of feature information from sparse channels while preventing overfitting.
3) A multi-stream fusion network based on CNN + GRU is designed to ensure accuracy while reducing the calculation time, making it more suitable for application in bionic hand control systems.

## Related Work

With the deepening of sEMG detection technology and the rapid development of computer technology, sEMG controlled human-machine interaction (Sun et al., 2020a; Chen et al., 2022c) systems can analyze the sEMG generated during the user’s movement to obtain the human body’s intention, and eventually control peripheral devices by transmitting movement commands (Pinto and Gupta. 2016; Zhang et al., 2019; Li et al., 2021; Liu et al., 2022a). Early prostheses were generally single-degree-of-freedom robotic arms with grasping capability only. Reitert (Scott, 1989) first used sEMG signals for prosthetic control in 1948, that’s the earliest. Carrozza (Carozza et al., 2005) implemented a single-degree-of-freedom sEMG prosthetic hand control (Andreas et al., 2017; Sun et al., 2020b; Liu et al., 2022b) using a finite state machine. Many researchers have worked on the problem of the multiclassification of sEMG. Traditional machine learning algorithms (Yu et al., 2019; Chen et al., 2022b) need to extract time-domain, frequency-domain, or time-frequency-domain features from sEMG data and select appropriate classifiers, such as extreme learning machines (Shi et al., 2013; Sun et al., 2022b), random forests (Atzori et al., 2014a; Asif et al., 2017), linear discriminant analysis, and support vector machines (Chen and Zhang, 2019), to accomplish the gesture recognition task. However, traditional machine learning tends to decrease the recognition accuracy significantly with the increase of gesture movements. From machine learning to deep learning (Lenz et al., 2015; Qi et al., 2020; Huang et al., 2021; Tao et al., 2022), the accuracy of the multi-classification of EMG signals has been improving. Deep learning performs better in the multi-classification problem of sEMG gestures because of its strong data fitting and feature extraction ability (Li et al., 2019c; Sun et al., 2022a). In the method of gesture recognition using deep learning (Han et al., 2018; Jiang et al., 2019b; Liao et al., 2021), there are several major types of mainstream network algorithms: 1) convolutional neural networks (Tao et al., 2022; Sun et al., 2022c); 2) recurrent neural networks (Liu et al., 2022b); 3) network combining multi-class models (Zhao et al., 2022); 4) some novel network (Huang et al., 2019; Chen et al., 2021b; Sun et al., 2021; Liu et al., 2022c; Liu et al., 2022d; Wu et al., 2022; Zhao et al., 2022).

In terms of EMG gesture recognition, CNN-based deep learning algorithms (Shi et al., 2022) have proven to be highly advantageous by many researchers (Huang et al., 2020; Jiang et al., 2021a; Yang et al., 2021)). Manfredo et al. (Manfredo et al., 2016) found that the average result of recognition accuracy of classical classification methods is easily surpassed by a simple CNN structure on the Nina Pro database. P. Tsinganos (Tsinganos et al., 2018) et al. used CNN networks for gesture recognition and improved the accuracy by 3%. Yu Hu (Hu Y. et al., 2018) et al. proposed an attention-based hybrid CNN-RNN (Recurrent Neural Network) model to process Nina Pro DB1, Nina Pro DB2, and Bio Pat Rec-26MOV compared to the normal hybrid CNN-RNN model databases, the accuracy was improved by 2.0%, 7.4% and 1.6%, respectively. Yuru Chen et al. use MYO hand ring to acquires upper limb EMG signals for data preprocessing, classification, and identification followed by real-time control of upper limb mechanical devices. Migratory learning, long and short-term memory networks, and recurrent neural networks were applied to EMG signal gesture recognition (Naik et al., 2014; Côté-Allard et al., 2017; Ding et al., 2018; Quivira et al., 2018; Xie et al., 2018; Chen et al., 2020), and Tsinganos et al. (Zardoshti-Kermani et al., 1995) researchers treated EMG signal-based gesture recognition as a sequence classification problem and introduced temporal convolutional networks for gesture recognition, with an improvement in recognition accuracy of about 5%. In general, machine learning methods for sEMG gesture recognition require low training data set size and short training time, but the requirements for researchers are relatively high; while deep learning-based methods have low or basically no requirements for feature set selection and certain requirements for sample data volume, because insufficient data will lead to poor recognition accuracy. We will combine attentional mechanisms and long short-term memory networks. Consider the sEMG signal as an image classification problem and time series classification as the basis for network design (Li et al., 2019b; Luo et al., 2020), establish a new network architecture for gesture recognition, further explore the optimization of network models, improve the recognition accuracy, and solve the problems of relatively long computation time, high hardware requirements in use, and unsuitable for the application of bionic The problem of relatively long computation time, high hardware requirements in use, and unsuitable for the application of the actual control process of bionic hands (Xiao et al., 2021; Liu X. et al., 2022).

## Methods and Materials

sEMG signal is a signal with temporal order (Luo et al., 2020), and RNN has shown excellent prediction capability in dealing with time series problems. GRU (Chung et al., 2014) is a further development based on RNN that can solve the problems of long-term learning dependence and long-term preservation of RNN and avoid gradient disappearance. The speedy, lightweight Conv1D combined with sequential-sensitive GRU to build the model can balance accuracy and speed, with fewer training parameters and faster convergence and iteration, which is beneficial to real-time recognition. Conv1D serves as a pre-processing step for GRU to shorten the identified sequences and extract local information before GRU processes the timing-related information.

### MCBAM-GRU Net Architecture

**FIGURE 1:**
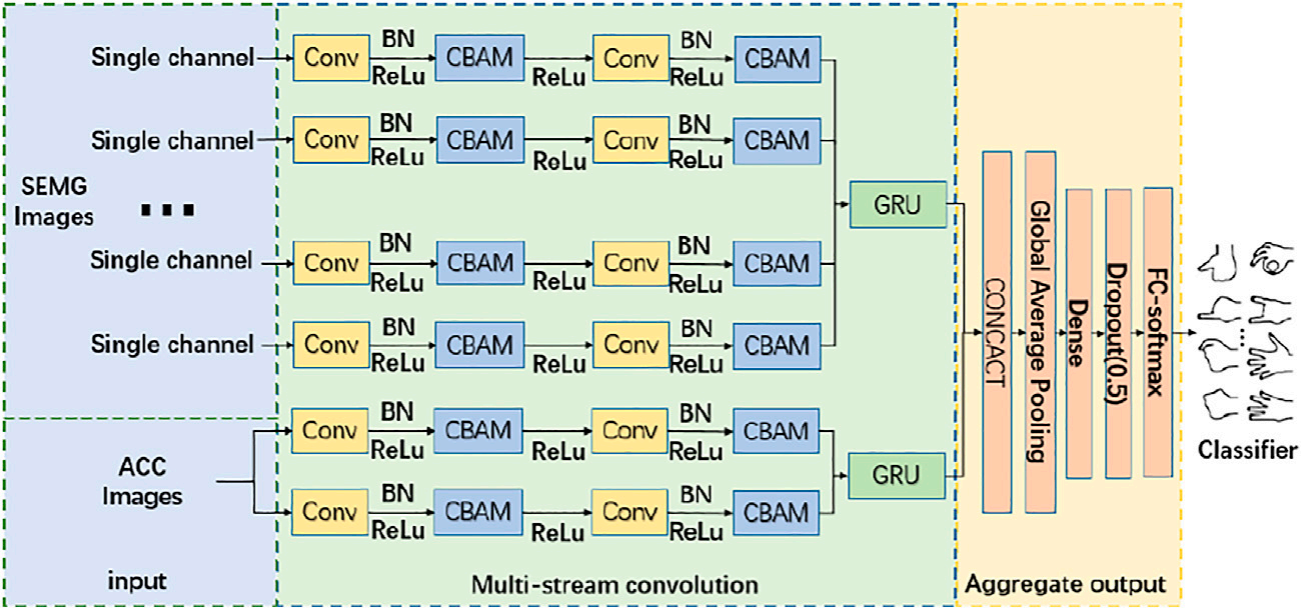
MCBAM-GRU general framework.

A multistream CNN is proposed, which fusing attention mechanism and long short-term memory network, called MCBAM-GRU network, whose general framework is shown in Figure 1. After supervised training, end-to-end gesture recognition of surface EMG signals can be performed. The overall model can be divided into three stages, the data input, the multistream convolution and the aggregated output.

First, the data input phase divides the EMG signal by acquisition channel dimension to obtain a single-channel EMG map. The multistream convolutional network (Sun et al., 2020c; Tran and Lin, 2021) has six independent network branches. The features of the EMG signals of the six channels are extracted separately for each stream. Meanwhile, to improve the recognition effect, the sEMG and acceleration are fused by information fusion technique (Sun et al., 2020a; Liao et al., 2020; Tian et al., 2020; Hao et al., 2021a; Jiang et al., 2021b; Bai et al., 2022; Huang et al., 2022), and the signal surface EMG and ACC signals are used as the input of each stream of independent CNN, and each stream learns features independently by MCBAM. Since the single-channel sEMG map is essentially one-dimensional time-series data, a one-dimensional convolution kernel is used in the batch normalized convolution layer to learn the hidden correlation between sEMG sequence signals.

The aggregation output stage aggregates all the outputs of the multi-stream convolution stage and obtains the final recognition results. The first layer is the aggregation layer, which aggregates the outputs of the multi-stream convolution using a cascade stitching of feature channel dimensions. The second layer is a global mean pooling layer, which averages all pixel values of the feature map to obtain a value. The third layer is a full connected layer with dropout, which reduces the dimensionality of the output vector and adds dropout to prevent overfitting. The final layer uses a softmax-activated fully-connected layer to obtain the classification results. This layer first obtains a label vector g of the length of the number of gesture categories through the fully-connected layer and subsequently uses a softmax function to predict the category to which the label vector g belongs.

$$p\left(m|g\right)=\frac{\exp\left(g\left(m\right)\right)}{\sum_{j=1}^{M}\exp\left(g\left(j\right)\right)}$$ (1)

Where M represents the total number of gesture categories; p(m|g) represents the probability that the vector g belongs to the Class m gesture, and the final category with the highest probability is the classification result $m_{0}$ .

$$m_{0}=\arg\max\left(p\left(1|g\right),p\left(2|g\right),\cdots ,p\left(M|g\right)\right)$$ (2)

### Redundant Channel Removal

For the region of EMG signal distribution studied in the experiment, the sEMG signals generated by different hand movements of muscles some distributed in forearm muscles have a certain regularity, that is, some muscles in do not produce useful signals or redundant information channels, these regions not only interfere with the classification, and will increase the amount of data and increase the computational burden, thus affecting the classification speed.

Therefore, a method of removing redundant channels is necessary (Sun et al., 2020b), which takes the arm without movement as a benchmark and takes the variance of the signal values of different actions to represent the degree of signal redundancy.

**FIGURE 2:**
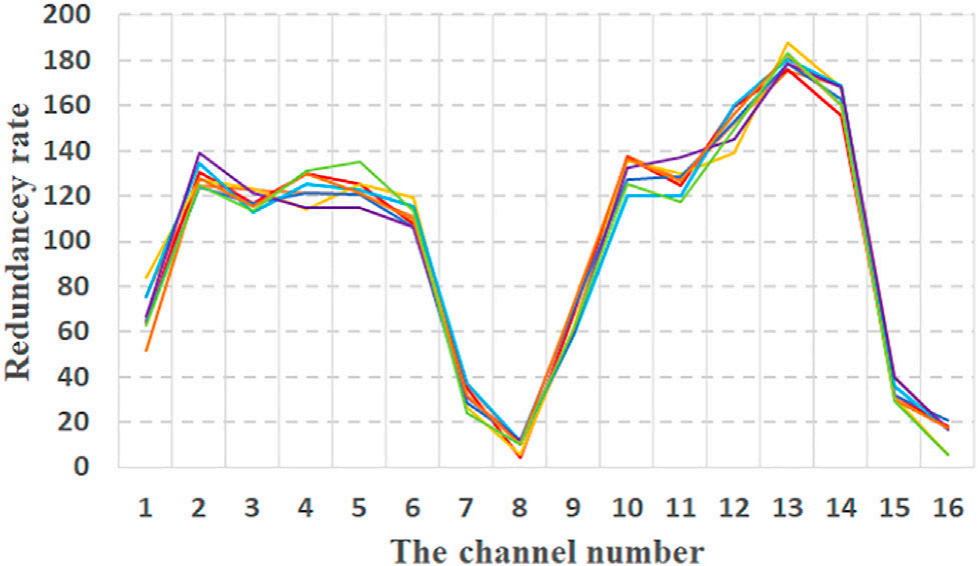
Weighted channel redundancy.

By using the above variance calculation values (Zhou et al., 2010) to grade the redundancy of 16 channels in each action. Simple sequencing coding methods are used to assign positive correlation weights to different levels. In order to ensure the stability of the method, the cross-validation method (Scheme and Englehart, 2011) is used to verify. The collected data is divided into eight parts. One of them is taken out in turn and tested in the remaining data. Figure 2 shows the redundancy rate of 16 channels obtained after eight tests.

According to the weighting result, with high redundancy rate ten common redundant channels 2, 3, 4, 5, 6, 10, 11, 12, 13 and 14, channels are removed.

### One Dimension Convolutional Block Attention Module

**FIGURE 3:**
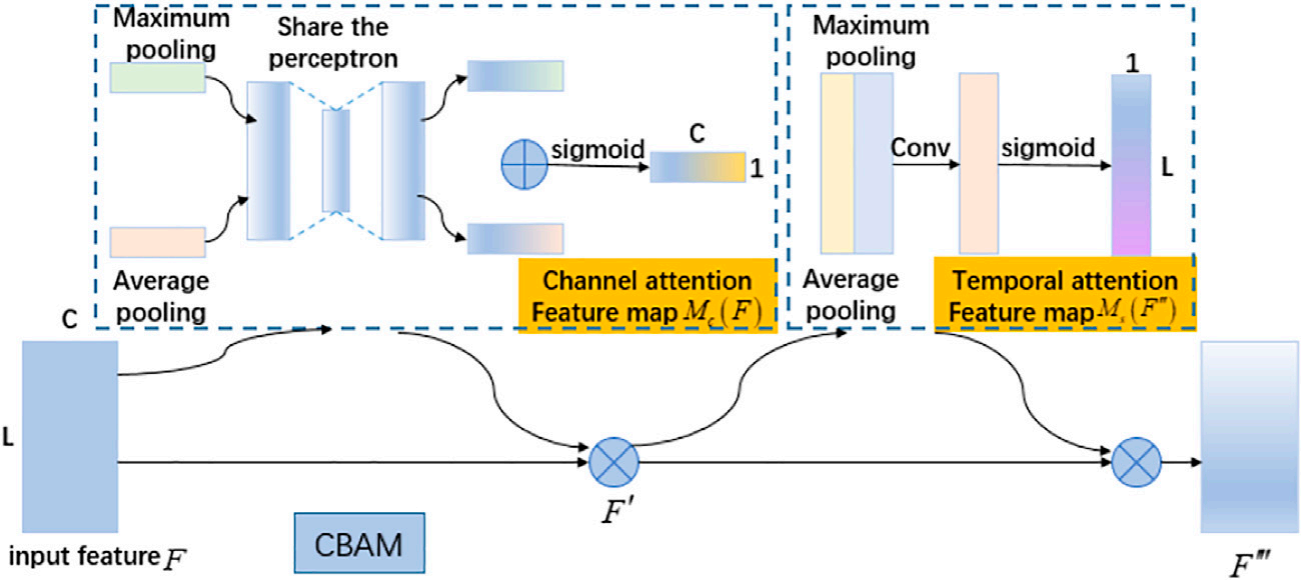
One-dimensional convolutional block attention module.

Attentional mechanisms (Hao et al., 2021b) has aroused the interest of many researchers because it have fewer parameters, faster speed, and better results in important areas such as machine translation (Hao et al., 2021a), speech recognition (Bahdanau et al., 2016; Rogowski et al., 2020), image recognition (Wang et al., 2017), and gradually started to be applied in sEMG gesture recognition (Hu J. et al., 2018; Jiang et al., 2019c; Liu et al., 2021) Previous CNN gesture recognition models often do not give enough attention to the characteristics of the EMG signal and do not make full use of the temporal information (Atzori et al., 2014b; Atzori et al., 2016; Geng et al., 2016; Ketykó et al., 2019). Therefore, this paper introduces a convolutional attention mechanism into the EMG gesture recognition method and designs a one-dimensional convolutional attention module based on time and feature channels to make it more applicable to EMG gesture recognition. A one-dimensional CBAM is added after the ReLU nonlinear function, which can redistribute the weights of EMG signals in different time frames and adaptively adjust the weights of each feature map to focus more on the effective features and suppress the useless features to some extent. The CBAM1D shown in Figure 3 is divided into the feature channel attention module and temporal attention module.

The specific main process of the feature channel attention module shows as follows: an input feature $F\in {\mathbb{R}}^{T\times C}$ along the time dimension to perform feature compression, a real number is used to represent the time dimension information. Temporal information is aggregated through the average pooling and maximum pooling. Generated two different feature vectors are $F_{b}^{C}\in {\mathbb{R}}^{1\times C}$ and $F_{n}^{C}\in {\mathbb{R}}^{1\times C}$ . The formula is shown below.

$$\begin{array}{l} F_{b}^{c}\in \frac{1}{T}\sum_{i=1}^{T}F^{c}\left(i\right)\\ F_{n}^{c}=\max\left(F^{c}\left(i\right)\right)\;i=1,2,\cdots ,T \end{array}$$ (3)

Among them: $F_{b}^{C}=\left[F_{b}^{1},F_{b}^{2},F_{b}^{3},\cdots ,F_{b}^{C}\right]$ ; $F_{n}^{C}=\left[F_{n}^{1},F_{n}^{2},F_{n}^{3},\cdots ,F_{n}^{C}\right]$ .

The two feature vectors are sent to a shared network to generate two new feature vectors, which are combined using element-by-element summation to generate the feature channel attention map $M_{C}\in {\mathbb{R}}^{1\times C}$ . The shared network consists of a two-layer multilayer perceptron. ℝCr×1 is the activation size of the first layer, which can reduce the number of parameters, where r is the compression rate; The second layer output size is ${\mathbb{R}}^{C\times 1}$ . The feature channel attention module can be expressed as shown in Eq. 4.

$$M_{C}\left(F\right)=\sigma_{g}\left(W_{1}\left(W_{0}\left(F_{b}^{C}\right)\right)+W_{1}\left(W_{0}\left(F_{n}^{C}\right)\right)\right)$$ (4)

where: $\sigma_{g}$ is the sigmoid activation function; W0∈ℝC×Cr ; W1∈ℝCr×C .

The spatial attention module uses the time-series relationship of the myoelectric map to generate the temporal attention map $M_{T}\in {\mathbb{R}}^{T\times 1}$ . Feature channel attention focuses on the effective feature maps, while temporal attention focuses more on the effective time frames in the decision window, which further complements the feature channel attention. The specific process of the temporal attention module is: feature compression along the feature channel dimension, using a real number to represent the feature channel dimension information of the feature. The average pooling and maximum pooling are used to aggregate the feature channel information to obtain two feature vectors, $F_{b}^{T}\in {\mathbb{R}}^{T\times 1}$ and $F_{n}^{T}\in {\mathbb{R}}^{T\times 1}$ .

$$\begin{array}{l} F_{b}^{t}\in \frac{1}{C}\sum_{j=1}^{C}F^{t}\left(j\right)\\ F_{n}^{t}=\max\left(F^{t}\left(j\right)\right)\;j=1,2,\cdots ,C \end{array}$$ (5)

Among them: $F_{b}^{T}=\left[F_{b}^{1},F_{b}^{2},F_{b}^{3},\cdots ,F_{b}^{T}\right]$ ; $F_{n}^{C}=\left[F_{n}^{1},F_{n}^{2},F_{n}^{3},\cdots ,F_{n}^{C}\right]$

Two feature vectors are cascaded and stitched by feature channel axes to generate a one-dimensional temporal attention map $M_{T}$ through a standard convolutional layer. Temporal attention module can be represented by Eq. 6.

$$M_{T}\left(F\right)=\sigma_{g}\left(f^{3\times 1}\left(\left[F_{b}^{T};F_{n}^{T}\right]\right)\right)$$ (6)

where: $\sigma_{g}$ is the sigmoid activation function; $f^{3\times 1}$ represents the convolution kernel is a 3*1 one-dimensional convolution.

Combining the time and feature channel attention modules, the total process can be summarized as follows

$$\begin{array}{l} F^{\prime }=M_{C}\left(F\right)⊗F\\ F^{\prime \prime }=M_{T}\left(F^{\prime }\right)⊗F^{\prime } \end{array}$$ (7)

where: ⊗ represents the element-by-element multiplication; $F^{\prime \prime }$ is the final output of the 1D CBAM module, the input features after remapping.

### EMG Signal Acquisition

The experiments use the ELONXI EMG instrument produced by Hangzhou Jiaopu Technology Company as the measurement sensor. ELONXI electromyograph has a 16-channel sEMG signal sensor. The sampling frequency is 1,000 Hz. This electromyograph has a high sampling frequency and is easy to wear, simple to use and low cost. This device is convenient for data acquisition and uses with dexterous hand mechanical devices. Before the experiment, the surface of each electrode of the cuff and the skin surface of the volunteer were gently wiped with alcohol cotton. Volunteers need to wait a few minutes for the skin surface to dry naturally and put the cuff on the forearm. The 16 electrodes of the cuff were numbered and evenly distributed on the surface of the arm. The correspondence between 16 electrodes and forearm muscles is shown in Table 1.

**TABLE 1:** The correspondence between 16 electrodes and forearm muscle.

Electrode Number	Strong Related Muscles	Weak Related Muscles
Electrodes 1 and 9	Finger deep flexors	
Electrodes 2 and 10	Ulnar carpal flexor	Finger deep flexors
Electrodes 3 and 11	Superficial finger flexors	Palmaris Longus
Electrodes 4 and 12	Radial wrist flexor	Palmaris Longus
Electrodes 5 and 13	Brachioradialis	
Electrodes 6 and 14	Radial wrist extensors	
Electrodes 7 and 15	Finger extensor muscle	
Electrodes 8 and 16	Ulnar carpal extensor	Little finger extensors

This experiment was approved by the Research Ethics Committee of Wuhan University of Science and Technology of China. Before the experiment, the relevant content of the experiment and the risks have been informed in detail to the 10 healthy subjects. And then they have signed informed consent forms. The experimental environment was quiet and free of absolute noise, Subjects seated in a chair where their hands can comfortably be placed on the table. They were asked to perform the corresponding right-handed movements according to the cues on the computer screen.

**FIGURE 4:**
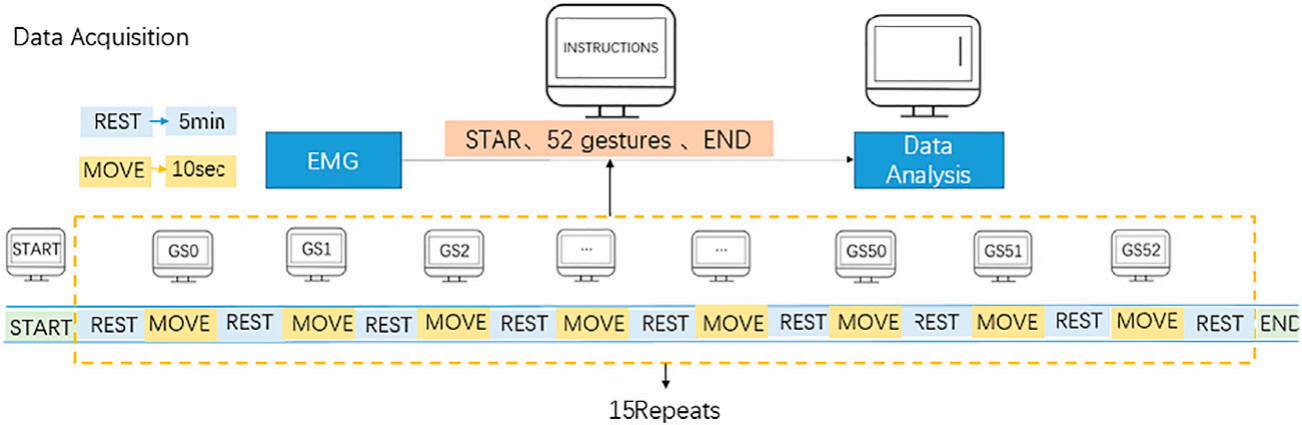
Timing diagram of EMG acquisition experiment.

Volunteers should try to maintain the same speed and force, with the muscle relaxation state as the initial state and each target movement as the end state of one movement. To ensure that there is a long enough interval between the two movements to avoid muscle fatigue. A total of 10 adult healthy subjects (7 males and 3 females) with no history of disease and a homogeneous physical distribution underwent forearm sEMG collection experiments, with an age distribution between 25 and 30 years old, all using the right arm. Each subject was required to acquire 52 gestures, with each action repeated 15 times, each action lasting 10 s, with a 5-min rest period between each action (the electrode cuff should not be removed during the experiment), to obtain the same body EMG signal with temporal and spatial differences in the EMG signal. During the acquisition process the subject followed the screen prompts, concentrated on the instructions on the screen, and the timing diagram of the EMG acquisition experiment is shown in Figure 4.

In previous studies, only 4–12 different movements were generally considered, but for a prosthetic manipulator, 4–12 gestures are far from sufficient. In this paper, 52 gestures from the NinaPro DB1 dataset were selected. These gestures are divided into three exercises. 1) 12 finger-based gestures, 2) eight equal-open, equal-length gestures and nine wrist-based gestures, and 3) 23 basic grasping gestures. 52 gestures are numbered as Gs1-52.

### Feature Extraction and Selection

The reasons and methods of sEMG signal preprocessing have been systematically summarized by many domestic and foreign EMG research results (Woo et al., 2018). The original image is replicated in layers to obtain the image of each channel, and then preprocess and normalize each channel sEMG before input to the neural network.

sEMG signals are extremely weak and susceptible to environmental noise, industrial frequency interference and individual body differences, and that leads to the signal-to-noise ratio of sEMG signals relatively low. To obtain a high signal-to-noise ratio and make the online system have good real-time performance, the sEMG collected is mainly distributed in the range of 0–500 Hz, with the main energy concentration in the range of 10–200 Hz. Considering the signal frequency, the sEMG may be interfered with by the 50 Hz industrial frequency interference and the low-frequency signal below 20 Hz. Therefore, a trap filter and a Butterworth high-pass filter are chosen for denoising.

The frequency response of the ideal trap filter is expressed as Eq. 8, where $\omega_{0}=50Hz$ , which is used to remove the 50 Hz industrial frequency interference.

$$\left|H\left(e^{jw}\right)\right|=\left\{\begin{array}{l} 1,\omega \neq \omega_{0}\\ 0,\omega =\omega_{0} \end{array}\right\}$$ (8)

The Butterworth filter amplitude and frequency should be shown in Eq. 9, where.

N represents the system order of the filter, N=3 , Ω represents the frequency, $\Omega_{c}$ represents the turning frequency. The selected high-pass filter with a frequency range above 20 Hz and an attenuation rate of 18 dB per octave.

$${\left|H\left(j\Omega \right)\right|}^{2}=\frac{1}{1+\varepsilon^{2}{\left(\frac{\Omega }{\Omega_{c}}\right)}^{2N}}$$ (9)

**FIGURE 5:**
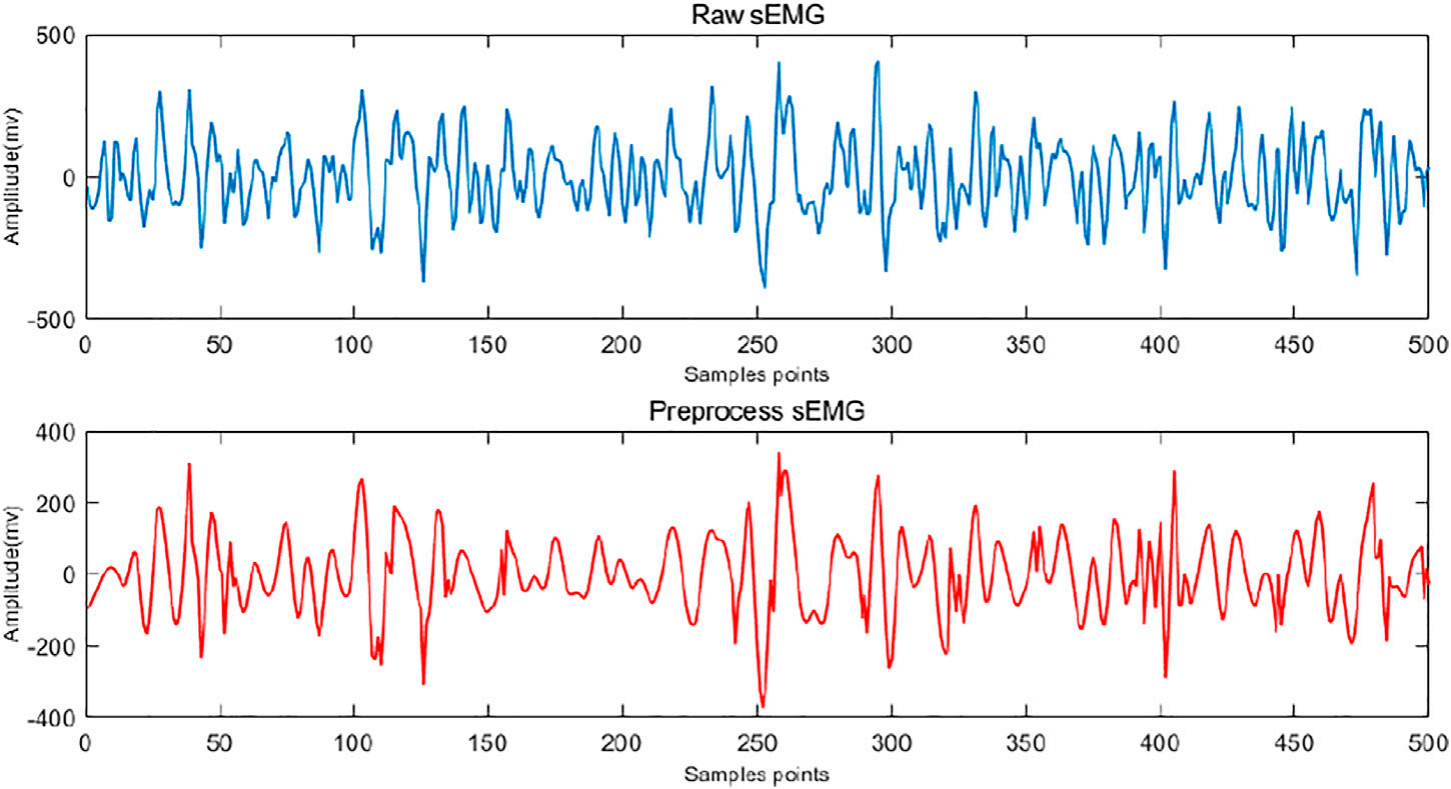
Visualization of sEMG waveforms before and after filtering.

The waveform and spectrum after pre-processing are shown in Figure 5.

Due to the small amount of energy produced by the target action muscles (Sun et al., 2020a; Cheng et al., 2020), to make the amplitude of the active segment more visible, an absolute value is taken for the acquired signal. sEMG signals are continuous signals, and if the whole segment of the acquired signal is used to characterize the target action. It is not conducive to the implementation of a real-time system and the classification of the target action. At the same time, the EMG signal has obvious non-smooth randomness, therefore, the pre-processed signal should be described by a set of data that can characterize its features, which can effectively distinguish different actions and facilitate classification.

Integral electromyogram (IEMG), myoelectric variance value (VAR), median frequency (MDF), and signal high-to-low frequency ratio (FR) are the four features selected in the experiments, calculated as Eq. 10, which are easy to calculate and high in real time to characterize the signal features. and the resulting image is used as a multichannel EMG feature image.

IEMG=∑i=1N|xi|     VAR=1N−1∑i=1Nxi2∑i=1MDFPi=∑i=MDFMPj=12∑i=1MPi FR=∑i=LLCLHCPi∑i=HLCHHCPi (10)

Where *x*_*i*_ and *P*_*i*_ represents the peak value of the *i*th point of sEMG in the time sequence; represents the peak value of the *i*th point of sEMG in the time sequence; *P*_*i*_ represents the power value of the *i*th point of sEMG on the spectrum; *N* represents the number of signal sampling points; *M* is the signal bandwidth. *LLC* and *LHC* are the lower and upper cut-off frequencies of the low-frequency band, respectively; HLC and HHC are the lower and upper cut-off frequencies of the high-frequency band.

## Experiment

The dataset is randomly divided into two groups: one is the training set, and the other is the test set. The training set contains 800 sets for each gesture, and each test set contains 100 sets. Experimental environment hardware: Intel(R) Core(TM) i7-9700K CPU@3.60 GHz; memory: 32.00 GB. All experiments are implemented by PyTorch 1.7.0 + cu110 on NVIDIA GTX 1080Ti GPU.

### Experimental Results and Analysis

**FIGURE 6:**
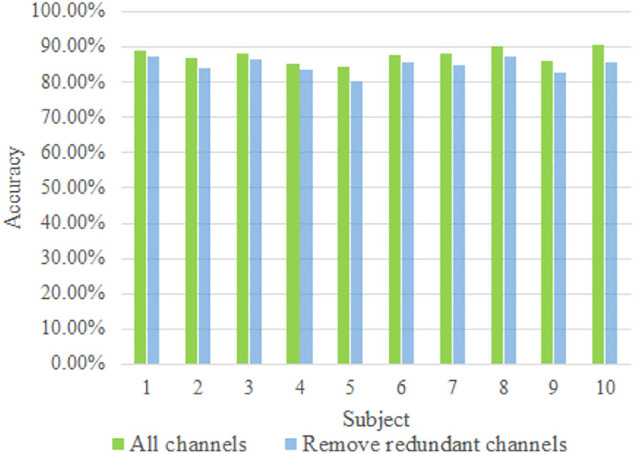
Removing redundant channels vs keeping all channels accurate.

During the experiment, redundant channels were removed to reduce the amount of computational data and to speed up the computational rate. Therefore, it is necessary to construct a comparison experiment between the sEMG signals after all channels were selected and the redundant channels were removed. In the comparison experiment, the network model was fine-tuned, and to prove its validity, no ACC information was added for fusion while the inputs were the original sEMG images. The comparison validation yielded the results shown in Figure 6.

By comparing the recognition rates of ten groups, the experimental results show that when the redundant channels are removed, the recognition efficiency does not change significantly, and considering the recognition rate and data dimension, it is necessary to remove the redundant channels.

Using the step-by-step debugging function of the Pycharm development environment, we input the action segment sEMG signal feature data from any test set into the trained network, and count the time from receiving data to output signal category as the delay time to measure the network computation speed. In order to exclude the chance influence of a certain input data, this paper brings in 10 times of data and calculates the average of network delay time. To ensure that the experimental results are not affected by the particular sEMG signal characteristics of a particular test subject, three subjects from the Nina Pro-DB1 database (Atzori et al., 2012; Atzori et al., 2014a) are selected for individual training and testing in this paper. The experimental results are shown in Table 2.

**TABLE 2:** Classification under removing redundant channels and keeping all channels.

Subjects	Channel selection	Delay (ms)	Testing Set Accuracy
Subject 1	All channels	82.5	85.3
	Removing redundant channels	64.3	82.2
Subject 2	All channels	76.1	78.1
	Removing redundant channels	61.9	76.6
Subject 3	All channels	80.8	87.0
	Removing redundant channels	63.8	84.2

From Table 2, it can be seen that removing redundant channels has lower latency and higher accuracy than keeping all channels. Therefore, removing the redundant channels with less data is more suitable for the application of the bionic hand control process.

To better validate the effectiveness of the MCBAM-GRU sEMG gesture recognition network, the following ablation experiments were performed using a matchless normalized multistream convolutional network as the baseline model for the five experiments. Raw sEMG images were used as the input source of the network and no ACC information was input for fusion.

Experiment I: multi-stream convolution.

Experiment II: multi-stream convolution + batch normalization (BN).

Experiment III: multi-stream convolution + batch normalization + Gate Recurrent Unit (GRU).

Experiment IV: multi-stream convolution + batch normalization + one-dimensional Convolutional Block Attention Module (CBAM).

Experiment V: multi-stream convolution + batch normalization + one-dimensional CBAM + Gate Recurrent Unit (GRU).

**FIGURE 7:**
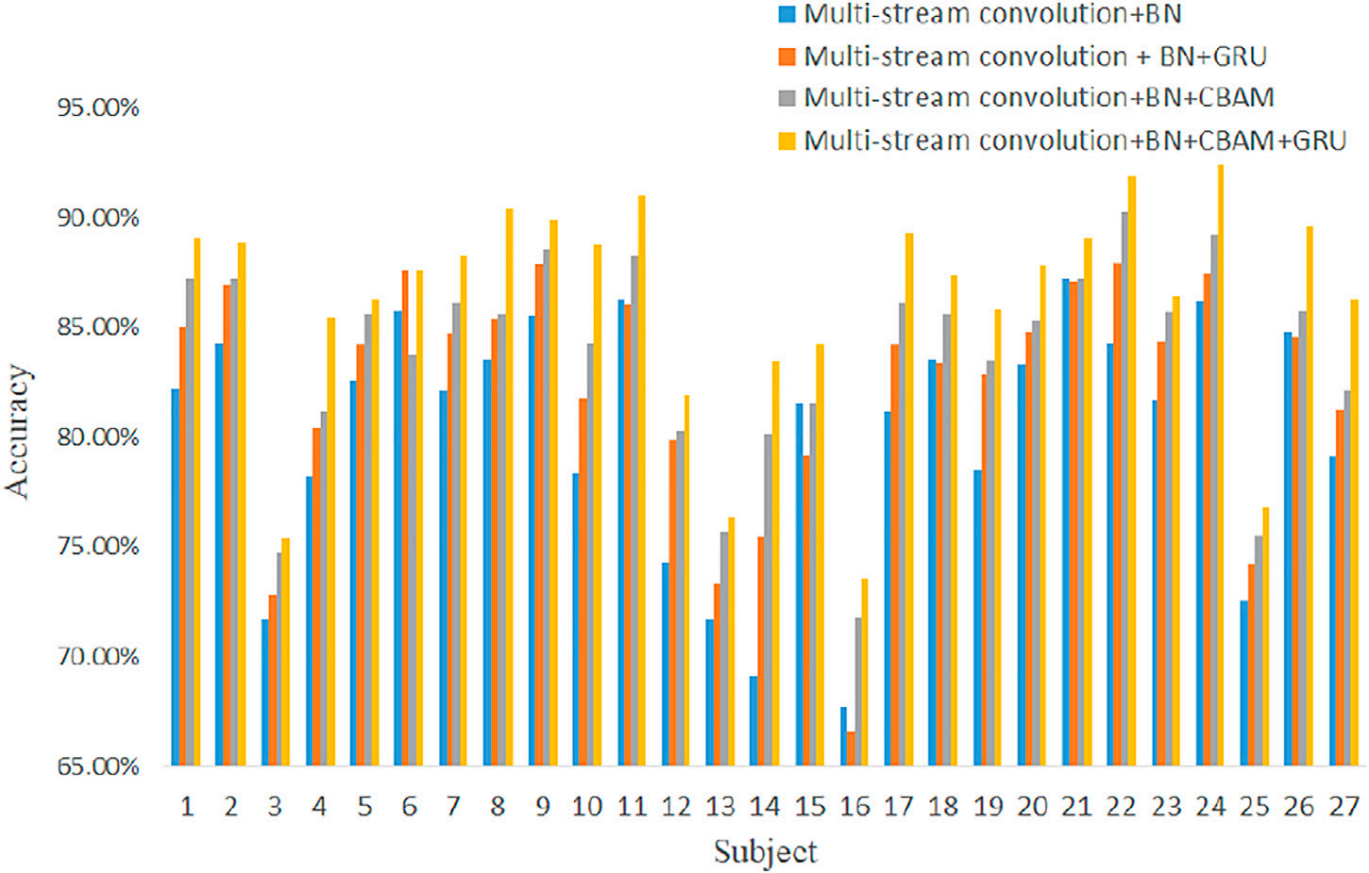
Results of 52 gesture recognition accuracy ablation experiments for each subject.

The results of the average gesture recognition accuracy ablation experiment are shown in Table 3. The results of the 52 gesture recognition accuracy ablation experiments for each subject are shown in Figure 7.

**TABLE 3:** Average gesture recognition accuracy ablation experiment results.

Method	Average Gesture Recognition Accuracy Rate/%
multi-stream convolution	68.3
+ batch normalization (BN)	80.2
+ BN + Gate Recurrent Unit (GRU)	82.1
+ BN+ 1D CBAM	83.6
+ BN+ 1D CBAM + GRU	86.0

From the experimental results in Figure 7, it is clear that the use of batch normalization has the greatest impact on recognition accuracy. The reason is that batch normalization can reduce the effect of internal covariate bias, reduce the sensitivity of different choices of parameter initialization and learning rate on the impact of model performance, and also facilitate gradient descent, which helps the model converge quickly. Therefore, adding batch normalization can help the network model fit the data set better and achieve higher performance. Adding one-dimensional convolutional attention to the multi-stream convolutional network can increase the average gesture recognition accuracy to 83.6% for 52 gestures for 27 subjects; adding GRU to the multi-stream convolutional attention mechanism network structure can increase the average gesture recognition accuracy to 86.0%. CBAM is introduced in the multi-stream network, which enables the network to learn saliency information in the image, making the important features in the image more salient and improving the expressiveness of the network without adding too many extra parameters and training time. The attention module generates temporal attention maps in the temporal dimension, giving more weight coefficients to the more important time frames in the time window and suppressing them with fewer weight coefficients on the contrary; in addition, it generates feature channel attention maps in the feature channel dimension, reinforcing the more effective feature maps and weakening the useless ones. The addition of the GRU module can further improve the network accuracy, enhance feature learning, and optimize network performance.

**FIGURE 8:**
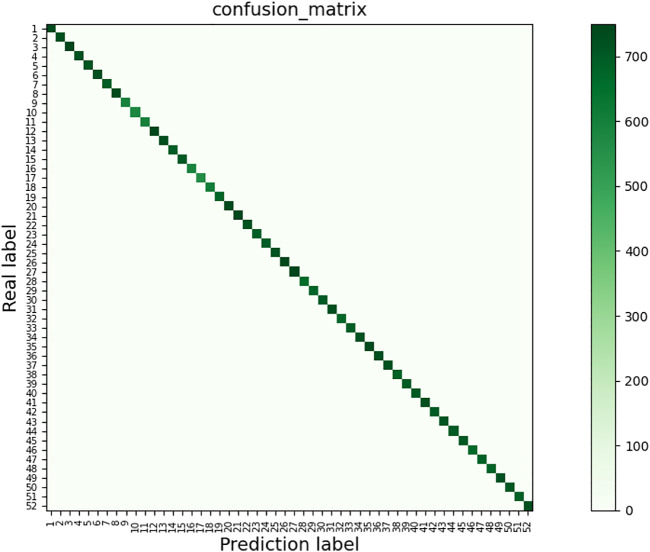
Gesture recognition confusion matrix.

Figure 8 shows the confusion matrix of the MCBAM-GRU network for gesture recognition, containing the prediction results for 52 gestures for 27 individuals. In this experiments, the acceleration (ACC) signal is input into the network as an independent branch, the characteristic EMG signal is the input source of the network, and other conditions remain unchanged. The horizontal coordinates are the predicted gesture labels and the vertical coordinates are the actual gesture labels. From Figure 8, we can see that the recognition accuracy of most of the movements are relatively high and are concentrated on the diagonal, but there are also individual gestures with low recognition accuracy, such as gestures 9, 10, 11, 16, and 17, which are easily misidentified because they are relatively similar. 9, 10, and 11 are all thumb movement gestures and have very similar force points, so the recognition is poor; 16 and 17 are also It is because the difference of only the direction of thumb movement is very similar, resulting in poor recognition.

To show the advantages of our model, MCBAM-GRU network recognition model is compared with other models that have been studied in recent years. The results on the NinaproDB1 dataset are shown in Table 4. The NinaPro DB1 dataset contains 52 different gestures of 27 healthy subjects, the same gestures contained in the dataset used in this paper. So we can compare the experimental results horizontally to conclude that our proposed MCBAM-GRU network algorithm outperforms other algorithms.

**TABLE 4:** Comparison results of different approaches on NinaPro DB1.

Algorithms	Accuracy (%)
Random Forest (Atzori et al., 2014b)	75.2
Atzori_Net (Atzori et al., 2016)	66.6
Geng_Net (Geng et al., 2016)	77.8
CNN (Du et al., 2017)	79.5
ELM (Cene et al., 2019)	75.1
MSFusionNet (Wei et al., 2019)	85.0
MCBAM-GRU (ours)	89.7

From the comparison of the results in the above table, it is obvious that with the application of deep learning on sEMG gestures, many researchers did not achieve better recognition results, and most of them did not exceed 80% recognition rate, which is only comparable to the recognition results of traditional methods. Except for the results in this Section, the recognition rate achieved by Wei’s proposed multi-stream convolutional neural network can reach 85.0%, while the multi-view fusion network proposed also can achieve a satisfactory recognition rate on the NinaPro DB1 dataset, which can reach 89.7%, 4.7% higher than the former.

### Validation Experiments

**FIGURE 9:**
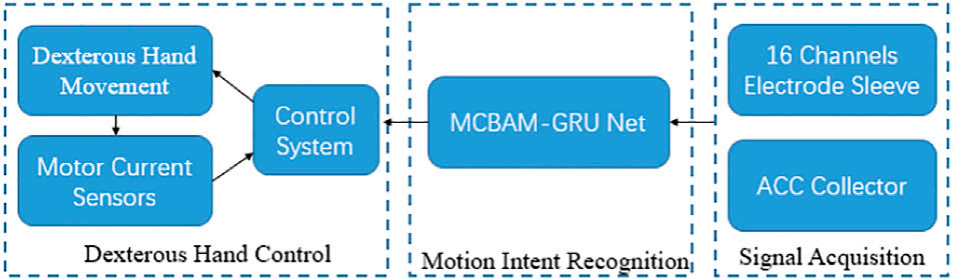
Block diagram of dexterous hand control system.

A 6-channel sEMG gesture recognition based dexterous hand control system is designed as a validation experiment platform, and the block diagram of the dexterous hand control system is shown in Figure 9. The system mainly contains three parts: sEMG signal acquisition, action intention recognition and prosthetic hand control. The control process of the dexterous hand is as follows: sEMG signal and acceleration acquisition equipment collects sEMG data from the user’s forearm in real time and sends it to the computer by wireless communication. The collected data is pre-processed and the redundant channels are removed as the input of the MCBAM-GRU network. The trained MCBAM-GRU network outputs the predicted current gesture, which is sent as the control signal of the dexterous hand to the dexterous hand console. Fifty-two bionic hand movements were preset in the console program, corresponding to 52 movements from the Nina-Pro-DB1 database (Atzori et al., 2016).

**FIGURE 10:**
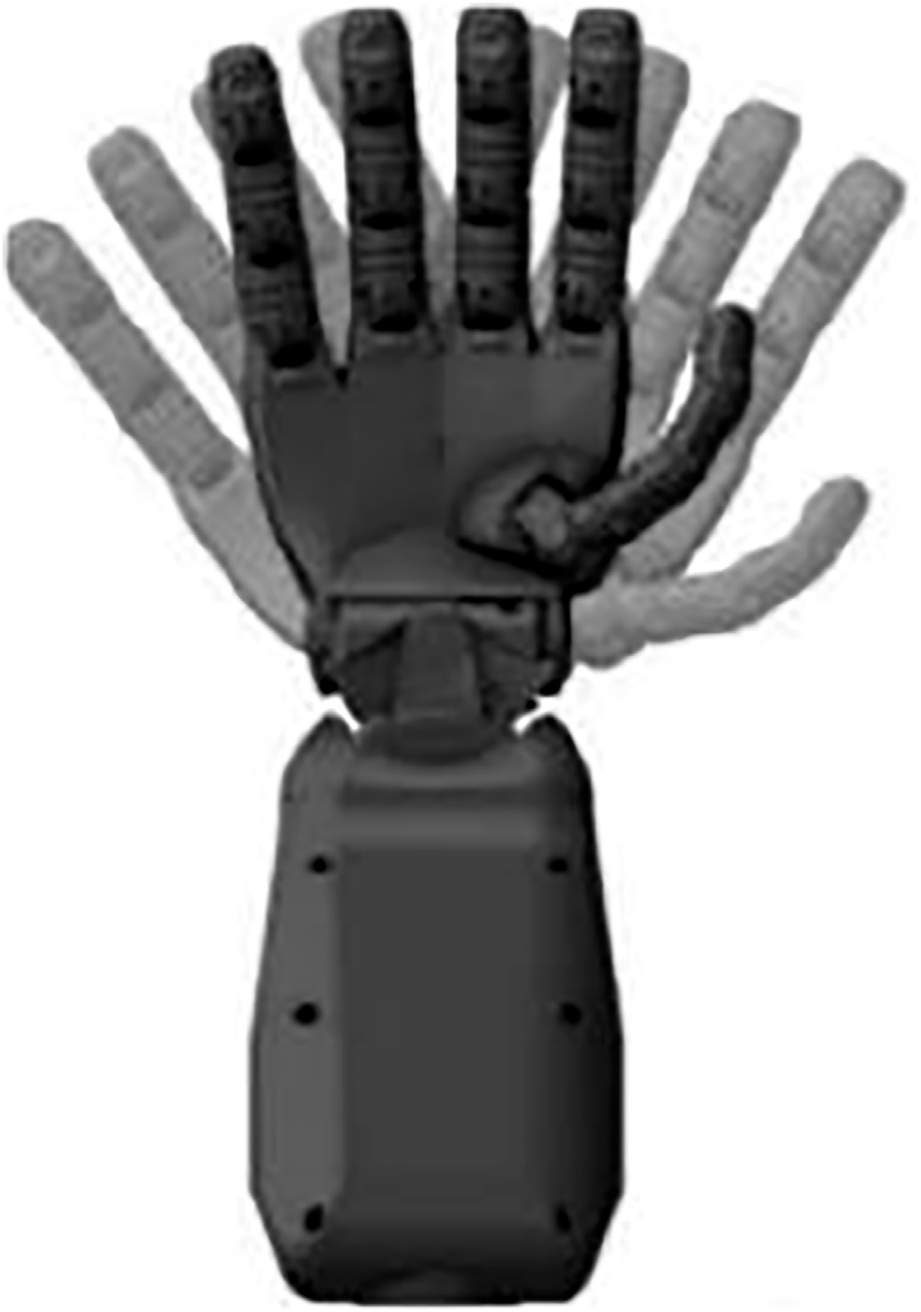
SR-RH8D humanoid dexterity hand.

The 16-channel sEMG sensor cuff can adapt to different users’ arm circumference and keep the electrode in good fit with the forearm; meanwhile, the sensor electrode is made of stainless steel, which is convenient to wear. The device connects the EMG collector to the computer via Bluetooth for fast data upload. The EMG cuff is worn on the right hand, and the accelerometer is placed close to the back of the hand to keep the forearm horizontal. The dexterous prosthesis used is an adult-sized anthropomorphic SR-RH8D (shown in Figure 10) developed by Seed Robotics in the United Kingdom. It is designed with underdrive technology, which allows the dexterous hand to be adaptive for precise control of objects with different or complex shapes.

The SR-RH8D has a total of 19 degrees of freedom, including the contralateral thumb and a full spherical wrist joint. In addition, it has eight compact and powerful internal actuators (seed_1-seed_8), all of which are contained within the unit and can be controlled independently by the user, and the relative relationship between the actuators and the dexterous hand motion is shown in Table 5.

**TABLE 5:** Servo and dexterous hand motion correspondence.

Servo Number	Function
Seed_1	Wrist rotation
Seed_2	Wrist swaying from side to side
Seed_3	Flexion of the wrist
Seed_4	Inward thumb
Seed_5	Thumb flexion
Seed_6	Index finger flexion
Seed_7	Flexion of the middle finger
Seed_8	Ring finger and pinky joint action

**FIGURE 11:**
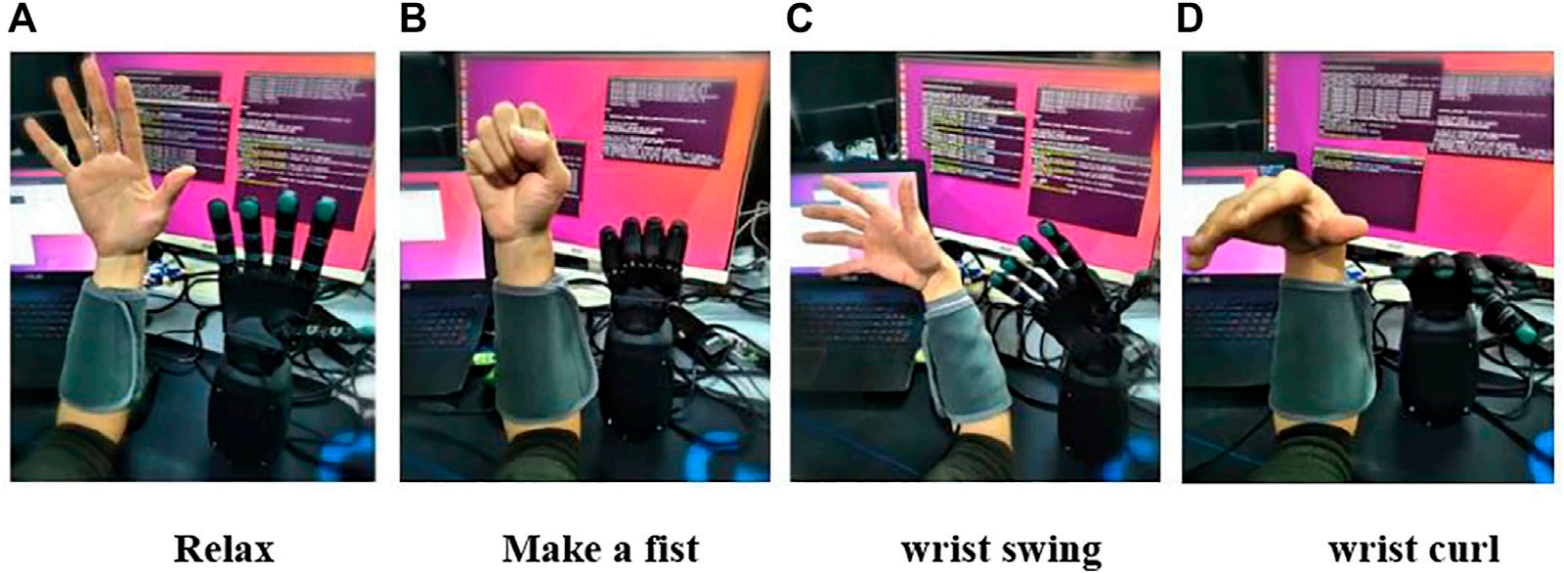
Experimental diagram of dexterous hand gesture movements (partial gesture demonstration).

The motor and control circuit of the dexterous hand are fully integrated into the dexterous hand, and which can be connected to a computer *via* a serial communication protocol. The trained multi-stream convolutional attention network is used to predict the gesture actions for the new input sEMG signals, and finally the predicted results are sent to the SR-RH8D dexterous hand as instructions to complete the corresponding gesture actions. The resting action was added to the experiment as a transition between the previous gesture and the next gesture action. The experimental results of some gestures are shown in Figure 11.

## Conclusion

Aiming at the insufficient effective feature extraction of sEMG timing information, the poor performance of gesture recognition speed and accuracy of sparse surface EMG signals, and the difficulty of application in dexterous hand control system, a multi-stream convolutional neural network MCBAM-GRU that integrates attention mechanism is proposed. it uses multi-stream convolution to excavate characteristics of the sparse channels of multiple acquisition channels. The one-dimensional convolutional attention module and the GRU module are added to learn important timing information and focus on more differentiated signal areas. Meanwhile the network adaptively learns the importance of different feature maps and strengthens the feature maps with stronger correlation, which ensure accuracy and greatly reduce the calculation time. The network can recognize 52 gestures using 6-channel surface electromyography and acceleration signals as model inputs. This multi-stream convolutional attention of this network can effectively prevent the overfitting problem of sEMG signals, while the use of GRU further improves the network accuracy. The prediction accuracy of the collected experimental data reached 94.1%, which is a satisfactory practicality. A dexterous hand control system is built to verify its feasibility. This network great potential for wider applications in future fields such as muscle fatigue and sensor electrode deflection. In subsequent studies, it is necessary to address differences in hand gestures, differences in arm size, and differences in the speed and strength of hand movements between individuals. There is a need for an in-depth study to obtain a pervasive multi-gesture recognition algorithm, Further simplify the network model, and broaden its application in resource-constrained embedded platforms.

## Data Availability

The raw data supporting the conclusion of this article will be made available by the authors, without undue reservation.
